# Beyond Awareness: Hope for a CMV Vaccine! An Introduction to the Conference, “CMV Vaccine Development—How Close Are We?” (27–28 September 2023) †

**DOI:** 10.3390/vaccines12111226

**Published:** 2024-10-29

**Authors:** Megan H. Pesch, Mark R. Schleiss, Stanley A. Plotkin, Sallie R. Permar, Rajeev Gautam

**Affiliations:** 1 Division of Developmental and Behavioral Pediatrics, University of Michigan, Ann Arbor, MI 48109, USA; pesch@umich.edu; 2 Division of Infectious Diseases, Department of Pediatrics, University of Minnesota Medical School, 2001 6th Street SE, Minneapolis, MN 55455, USA; 3 Perelman School of Medicine, University of Pennsylvania, 3400 Civic Center Boulevard, Building 421, Philadelphia, PA 19104, USA; 4 Department of Pediatrics, Weill Cornell Medicine, 1300 York Ave Box 65, New York, NY 10065, USA; sap4017@med.cornell.edu; 5 Program Officer at Virology Branch, Division of Microbiology and Infectious Diseases, NIAID, NIH, 5601 Fisher’s Lane, Rockville, MD 20892, USA; rajeev.gautam@nih.gov

**Keywords:** congenital cytomegalovirus (CMV), CMV vaccine, CMV advocacy, CMV immunity

## Abstract

Congenital cytomegalovirus (cCMV) is the most common infectious cause of disability in children. The major theme of this National Institute of Allergy and Infectious Diseases (NIAID) workshop, “CMV Vaccine Development—How Close Are We?”, was to report progress on the development of a pre-conception vaccine that could confer protective immunity for women of child-bearing age. Such a vaccine could result in a reduced cCMV disease burden, although other populations, including solid organ transplant and hematopoietic stem cell transplant patients, could benefit as well. To frame the compelling need for a cCMV vaccine, a keynote lecture by Dr. Megan Pesch, immediate past-president of the National CMV Foundation and a leading cCMV researcher from the University of Michigan, was given. This manuscript provides a summary of Dr. Pesch’s presentation from this workshop, which was written as the introductory conference report for the meeting.

## 1. Introduction

By way of introduction, this paper summarizes the content of a lecture, delivered by Dr. Megan Pesch, at a conference sponsored by the National Institute of Allergy and Infectious Diseases (NIAID) workshop held on 27–28 September 2023. The workshop, entitled “CMV Vaccine Development—How Close Are We?”, provided an overview of current progress toward the development of a cytomegalovirus (CMV) vaccine. However, although many populations would benefit from such a vaccine, a key theme in particular was the public health importance of cCMV infection. To emphasize the overall tone of the compelling need for a vaccine to enhance pregnancy outcomes, one of the keynote talks was delivered by Dr. Megan Pesch from the University of Michigan. This manuscript represents a summary of Dr. Pesch’s lecture.

## 2. Summary of Conference Presentation and Discussion

Dr. Pesch noted in her remarks that CMV has represented a formidable infectious adversary to vaccine developers over the last fifty years [1,2]. Most often silent, the virus can also cast a long shadow over the lives of affected individuals and families [3]. It was emphasized that CMV is not a new virus, nor is CMV vaccine development a new pursuit. In fact, in 1999, CMV was assigned the highest priority target for vaccine development from the National Academy of Medicine, owing to the estimated economic costs and human suffering that would be alleviated by reducing the incidence of cCMV infection [4]. Nearly a quarter century later, in September 2023, The National Institute of Allergy and Infectious Diseases (NIAID) hosted this workshop of leading experts from academia, industry, advocacy, and federal agencies to exchange information about recent advances and challenges in the pursuit of a CMV vaccine, and the meeting proceedings are reported in a separate manuscript collated from the workshop. Dr. Pesch acknowledged that the sharing of insights from the current NIAID workshop regarding the state of CMV vaccines was a key theme of the meeting. The scientific summary of the presentations focused on the development of an effective CMV vaccine is presented in a conference report accompanying this paper. While the focus of the NIAID meeting was to report on the scientific progress in CMV vaccine development, Dr. Pesch noted during her talk that it was important to remember why this pursuit is important in the first place—in particular, to the families and individuals impacted by this virus. While industry and academia have been working towards developing a vaccine, many in the greater cCMV community, specifically families, have been laying the groundwork for enthusiastic acceptance of a vaccine candidate when one is brought to market. This groundwork includes heightened awareness, expansion of newborn cCMV screening, and public support of vaccine development initiatives.

It was commented by Dr. Pesch that in the last ten years, largely driven by public health awareness initiatives, we have seen steady increases in the rates of identification of cCMV infection. Significant advances in scientific knowledge surrounding cCMV have also been made. In spite of these advances, we still have not kept pace with the magnitude of the epidemic [5,6,7]. For non-profit organizations, such as the National CMV Foundation, the decade began with campaigns to raise awareness about CMV prevention among pregnant women, which, ironically, were led by mothers of children with cCMV. Mothers who, like Dr. Pesch, did not receive this information in pregnancy. Today, Dr. Pesch noted that these campaigns continue to be fueled by selfless volunteers, such the network of parents representing 30 states as National CMV Community Alliance Chairs, who raise awareness about CMV prevention and engender hope for the next generation. When last studied in a large US cohort, only 9% of women had heard of cCMV [8]. In virtually all surveys, respondents’ awareness of CMV is lower than all of the other conditions that can impair pregnancy outcomes, such as spina bifida, Down syndrome, and fetal alcohol syndrome [9,10]. There is an as-yet unmet need to regularly perform large-scale public awareness assessments to better understand if and how ground is being gained. Prevention studies for cCMV lag far behind the mobilization of resources marshalled for control of epidemics such as HIV and Zika—in fact, the only large-scale prevention study for cCMV in the last decade was a failed human immunoglobulin-based intervention [11], an approach which was quickly forgotten once the COVID-19 pandemic took hold.

Dr. Pesch stressed that the families of children with cCMV have worked tirelessly to make sure their stories are heard, and perhaps most importantly, that women deserve to know about CMV [3]. The voices of the cCMV community have protested what many view as paternalistic recommendations, promulgated by the American College of Obstetricians and Gynecologists (ACOG), against educating pregnant people about the risks of CMV, assuming behavioral change during a pregnancy would likely be “impractical” and “burdensome” to implement [12]. It was asserted that women deserve to know the facts about their own bodies and the risks to their pregnancies. The point was stressed that the risk of cCMV, which impacts 1 in 200 live births [13], does not go away with silence. Awareness efforts also need not be limited to women of childbearing age, but also the village of people surrounding them, including their partners, public health officials, healthcare providers, and government health agencies. With recent innovations in CMV diagnostics [14,15] and vaccines [16], and with a heightened understanding about the potential lifelong impact of cCMV [17,18,19], an appeal was made to the audience that all individuals invested in the study of CMV should work together to expand shared goals to extend beyond raising awareness to enacting policies that result in behavioral changes that directly prevent the infection from occurring in the first place.

On a personal note, Dr. Pesch shared that her family had been touched by cCMV. It was noted that for those personally affected, feelings about the virus and even a potential vaccine may be complex [3,20]. For some women, cCMV infection has caused pregnancy loss, or taken the lives of their children [21], which is unequivocally tragic. For those who have living children impacted by prenatal acquisition of CMV, such individuals may feel conflicted about CMV and the disabilities that can come with congenital infection [3]. As a parent of a disabled child, Dr. Pesch commented that it was impossible to imagine her daughter without her autism and hearing loss. In fact, some of the most endearing aspects of her daughter’s personality were because of these “conditions”. She noted that her daughter’s disabilities were not, in and of themselves, the key issue: rather, the issue was the in utero insults caused by the CMV virus, which include the invasion of the fetal central nervous system and an attack on the placenta, that in turn led to life-altering consequences for her daughter and her family. To be clear, Dr. Pesch asserted that for her and other affected families, it was CMV that was the problem—and not their precious children.

It was pointed out that, in years past, the number of infants with diagnosed cCMV represented fewer than 5% of the total number of cases, due to the often invisible nature of the infection [22,23]. However, diagnosis is becoming increasingly common, likely due to newborn screening programs which have been growing in number and acceptance as a result of advocacy by affected families, starting with Utah’s pioneering legislation in 2013 [6]. With early diagnosis comes the opportunity for early treatment for all infants [24]. Treatment may include options such as anti-viral therapy (for those that will benefit from the drug) and, importantly, may also include early intervention services, audiologic monitoring, anticipatory guidance, and developmental supports. It was stressed during the lecture that, in addition to the enhancements in science supporting the efficacy and acceptability of a vaccine candidate, consideration needed to be given to vaccine acceptance. This will be optimized (1) if individuals have heard of the condition (cCMV); and (2) if healthcare providers who make vaccine recommendations are able to confidently describe the potential sequelae that could be avoided in future offspring by maternal vaccination. Led by Minnesota, several other states have also implemented state-wide universal cCMV screening, which may also prove critical for increasing both CMV awareness and future vaccine acceptance [25]. Universal screening will serve not only to allow an opportunity for treatment, but will also shed light on the majority of cases of cCMV that would have otherwise gone unrecognized. Dr. Pesch noted that, at present, most people are probably acquainted with an individual who has cCMV, although they may not be aware of the diagnosis. As neonatal diagnoses are facilitated and case recognition is enhanced, it was predicted that public awareness about the infection would grow. Improved case ascertainment will allow for more robust studies regarding the natural history of this highly variable condition, and perhaps the recognition of subtle, heretofore unrecognized long-term sequelae would drive further enthusiasm for licensure of a cCMV vaccine.

Dr. Pesch observed that the weight of carrying a CMV vaccine candidate from development to market need not be borne solely by researchers and industry. Other members of the public can and should engage in high-impact advocacy activities to contribute to this endeavor. Firstly, staying informed about CMV and its implications can help raise awareness and foster a sense of urgency for vaccine development. There is a need to continue to gauge the levels both of cCMV awareness and vaccine acceptance to ensure successful uptake of a CMV vaccine once it is launched. Additionally, participation in clinical trials, when available and as appropriate, aids researchers in their quest to gather valuable data. Diversity, equity, and inclusion will be key components of the assessment of a candidate vaccine’s safety and efficacy. Advocating for increased funding for cCMV vaccine research at both governmental and non-governmental levels is another powerful means of support. The amount of funding directed towards cCMV research is disproportionately low, given the public health impact of cCMV, and this deficiency must be rectified.

## 3. Concluding Remarks

In her concluding remarks, Dr. Pesch commented that the possibility of a CMV vaccine brings with it hope for future generations of children that may be born without the infection. Hope was also expressed that with a successful vaccine there would be a lowered burden of CMV-related complications for transplant recipients and possibly for those individuals with immunosuppressive conditions, including HIV infection. Dr. Pesch expressed her hope that in sixty years, her daughter’s grandchildren would reminisce with her about her childhood, looking back on her cCMV like an “old-timey, rarely-heard-of” infection. Through their work to enable an effective vaccine, she challenged the conference attendees to make CMV the next rubella, the next polio, the next diphtheria. By collectively recognizing the significance of CMV and actively contributing to the efforts of vaccine developers, Dr. Pesch noted that all researchers and advocates can play a vital role in advancing medical breakthroughs that benefit global public health.

Until such time as a vaccine is licensed, she urged all meeting attendees to keep up the work required to support this goal through recognizing the public health significance of cCMV, promoting newborn screening policies, and advocating for more research funding. Dr. Pesch referenced several of the vaccines in development that were highlighted at the conference, and these are described in greater detail in the paper accompanying this report [26]. Together, it was noted that we can ensure that the steady surge we have witnessed in scientific and public health momentum continues until a successful CMV vaccine is licensed, accepted, and widely used in the population.

Key Summary Points of Dr. Pesch’s Lecture:

• In addition to input from basic, clinical, and translational scientists, the greater cCMV community, particularly families, have been laying the groundwork for a vaccine for cCMV and can play key advocacy roles in moving vaccines forward.
• The National CMV Foundation, working nationally and regionally through its Community Alliance Chairs, provides valuable educational resources that help to close the knowledge gap about the impact of cCMV.
• Prevention efforts for cCMV have lagged far behind those developed for Zika and COVID-19, and this disparity needs to be addressed—particularly in light of the fact that preventative interventions are available and should be utilized even while vaccines are in development.
• ACOG should become more engaged in cCMV counseling, screening, advocacy, and vaccine research.
• A family’s personal story can carry a great deal of weight in moving vaccine research forward.
• Ultimately, the burden of moving a CMV vaccine candidate from development to market need not be borne solely by researchers and industry—the public can and should engage in high-impact advocacy activities to contribute to this endeavor.

## Data Availability

Original notes obtained during the conference are available upon request.
